# Adaptive multi-scale feature refinement for wheat phenology recognition using cross-scale attention mechanisms

**DOI:** 10.3389/fpls.2026.1730706

**Published:** 2026-03-19

**Authors:** Haifang Sun, Liang Hou, Xiaorui Guo, Yanan Wang, Jianan Min, Xiaoliu Zheng, Zhehao Tian, Donghui Zhang, Xinshi Zhang, Senhao Liu, Yu Gao, Zhaoli An, Hao Qi, Liangjie Lv

**Affiliations:** 1 Institute of Agricultural Information and Economy, Hebei Academy of Agriculture and Forestry Sciences (HAAFS), Shijiazhuang, China; 2 Institute of Cereal and Oil Crops, Hebei Academy of Agriculture and Forestry Sciences (HAAFS), Shijiazhuang, China; 3 Hebei Agricultural Technology Extension Station, Shijiazhuang, China; 4 Institute of Remote Sensing Satellite, China Academy of Space Technology (CAST), Beijing, China; 5 School of Microelectronics and Communication Engineering, Chongqing University, Chongqing, China; 6 Aerospace Information Research Institute, Chinese Academy of Sciences (CAS), Beijing, China; 7 Hebei Academy of Agriculture and Forestry Sciences (HAAFS), Shijiazhuang, China

**Keywords:** AMFR-Net, deep visual recognition, edge deployment, field-based RGB imagery, growth stage classification, multi-scale attention, precision agriculture

## Abstract

Accurate delineation of crop growth stages under real-world field conditions remains a long-standing challenge in computational phenotyping, particularly for wheat whose developmental phases are characterized by subtle, continuous morphological transitions and environmental noise. In this study, we propose AMFR-Net, an Adaptive Multi-Scale Feature Refinement Network tailored for fine-grained wheat stage identification using ground-level RGB imagery. Unlike conventional architectures that struggle with ambiguous inter-stage boundaries and rigid receptive structures, AMFR-Net leverages a ResNet-101 backbone augmented by a novel Adaptive Multi-Scale Attention Fusion (AMSAF) module—comprising cross-scale interaction blocks and confidence-weighted feature aggregation—to hierarchically recalibrate spatial–semantic representations. This design enables the network to adaptively amplify phenologically salient cues while suppressing irrelevant context, ensuring robust generalization under constrained annotation and deployment conditions. Evaluated on the expert-labeled CGIAR benchmark, AMFR-Net achieves state-of-the-art performance across all major metrics (Top-1 Accuracy: 89.10%; Macro-F1: 89.10%; AUC: 97.88%) and demonstrates superior discriminability in phenologically adjacent stages compared to lightweight and deep CNN baselines. Ablation studies validate the synergistic effect of multi-level attention and scale-aware refinement. The proposed framework offers a scalable, interpretable, and field-deployable solution for *in-situ* phenology monitoring, and sets a foundation for future integration of multimodal sensing, weak supervision, and cross-seasonal adaptation.

## 1 Introduction

Ensuring global food security under the dual pressures of climate variability and rising population demand hinges on our ability to monitor and optimize crop growth with precision (Fatchurrachman et al., 2022; Cui et al., 2023; Chen et al., 2024). Among the key levers of agronomic management, the timely and accurate identification of crop growth stages plays a central role, enabling irrigation (Mirshekarnezhad et al., 2018), fertilization (Hassanzadeh et al., 2020), pest control (Zhuang et al., 2022), and yield forecasting to be aligned with plant physiology. In large-scale production systems, however, traditional phenological observation remains constrained by labor intensiveness, subjectivity, and limited scalability (Ashourloo et al., 2022; Arun and Karnieli, 2024; Brinkhoff et al., 2024). With the advancement of image-based phenotyping and AI-driven decision systems, there is growing momentum toward automating stage recognition using ground-level RGB imagery (Kierdorf et al., 2023). Unlike satellite or UAV platforms that prioritize wide coverage, ground-level imaging offers high-frequency, low-cost observation at the canopy scale, which is attractive for both experimental and production settings (Li et al., 2022; Ni et al., 2024). Nonetheless, visual stage recognition faces challenges distinct from object detection or disease classification: phenophase transitions are gradual, visual cues overlap across adjacent stages, and field noise such as occlusion, uneven illumination, and growth heterogeneity further obscures boundaries—especially around mid-stage transitions such as booting to heading and heading to anthesis (Domingues et al., 2022; Memon et al., 2022; Camintia et al., 2024; Islam et al., 2025). Lightweight and interpretable models are also required for handheld and low-power sensors, yet conventional deep models may overfit scarce data or miss fine-grained differences due to rigid receptive structures (Zhou et al., 2024).

Recent progress in visual recognition has largely been driven by attention/Transformer-based backbones that improve long-range dependency modeling and global context aggregation, such as Vision Transformer (ViT), DeiT, and hierarchical designs like Swin Transformer. However, for field phenology recognition where training data are limited and deployment often targets edge devices, directly adopting large Transformer backbones may be suboptimal due to data hunger and computational overhead; meanwhile, modernized ConvNet families show that competitive performance can be achieved when convolutional priors are properly redesigned. These observations motivate a model that preserves CNN efficiency while injecting attention-driven cross-scale/global reasoning tailored to subtle phenological transitions.

Despite the importance of timely growth-stage recognition for field management, wheat phenology identification from ground-level RGB images remains difficult in practice because stage transitions are gradual and visually overlapping, while real-field acquisition introduces illumination, viewpoint, and occlusion noise. Meanwhile, real deployments often face limited annotations and require models that are accurate yet computationally feasible on edge devices. This combination creates a practical gap: conventional CNN classifiers may miss weak, distributed phenotypic cues due to rigid receptive structures, whereas Transformer-heavy solutions can be data-hungry and costly for field deployment. These considerations motivate us to develop a deployment-aware architecture that preserves CNN efficiency while introducing attention-driven cross-scale reasoning to enhance fine-grained stage discriminability.

### 1.1 Background and related work

Deep learning now underpins agricultural visual analytics across disease and pest detection, weed management, crop classification, and yield estimation. CNNs are particularly effective when tasks rely on high-contrast, localized features (Sarkar et al., 2023; Bhargava et al., 2024; Karim et al., 2024), detecting pests such as aphids and whiteflies (Pereira et al., 2022), and supporting weed management (Mckay et al., 2024). These studies primarily rely on high-contrast, localized visual features, which lend themselves well to CNN-based classification. Beyond plant pathology, CNNs have also been successfully applied to field-level phenotyping tasks such as fruit maturity detection (Maceachern et al., 2023), ripeness estimation, and animal health monitoring (Chelotti et al., 2024), highlighting their broader utility in precision agriculture.

Nevertheless, the limitations of purely CNN-based pipelines become more pronounced in fine-grained recognition problems where discriminative cues are subtle and spatially dispersed. Standard CNN classifiers typically rely on progressively enlarged receptive fields and global pooling to summarize features, which can dilute weak stage-specific signals and provide limited explicit mechanisms for long-range dependency modeling or cross-scale token interaction. In contrast, recent state-of-the-art backbones increasingly rely on self-attention to connect distant image regions and to selectively emphasize informative parts, including ViT/DeiT and hierarchical Transformers such as Swin. This trend is also evident in fine-grained visual classification (FGVC), where Transformer-based designs (e.g., TransFG) exploit attention maps to localize discriminative patches and improve separability between confusing subcategories. Despite their strong representational power, Transformer-heavy solutions may require more data and compute, which conflicts with real-field phenology settings that demand robustness under limited annotation and practical inference budgets (Bavana et al., 2024; Farooqui et al., 2024a, Farooqui et al., 2024b).

Despite these advances, the application of deep learning to phenological stage recognition remains comparatively limited. Unlike disease or pest detection, growth stage classification demands the modeling of continuous morphological transitions, where visual distinctions between adjacent stages are often subtle and ambiguous. For instance, the progression from vegetative to reproductive stages in cereal crops like wheat is characterized by gradual canopy changes, overlapping features, and environmental variability—conditions under which traditional CNNs often struggle to maintain discriminative performance. Existing works such as Jiang et al. (2021); Picon et al. (2019) have demonstrated multi-task and metadata-aware learning strategies for crop trait classification, yet these are primarily focused on discrete classes rather than temporally evolving growth phases (Picon et al., 2019; Jiang et al., 2021).

To address the fine-grained nature of phenological transitions, recent research has explored the integration of advanced network designs and learning paradigms. Transfer learning approaches using pre-trained models like ResNet, Inception, and DenseNet have been applied to tasks such as tomato and rice leaf disease classification (Sakkarvarthi et al., 2022), while meta-learning strategies have shown promise in adapting to variable image domains and limited annotation scenarios (Memon et al., 2022). Meanwhile, hyperspectral imaging combined with CNNs has been used to enhance the representation of subtle visual cues in crop phenotyping (Bhatti et al., 2023), though such modalities often require specialized hardware and are not always field-deployable.

Recent Advancements in Fine-Grained Phenology. The field has recently seen specialized architectures targeting the subtleties of crop growth. For instance, Ni et al. (2024) proposed a hybrid model combining CNNs with self-attention mechanisms to capture global dependencies in maize growth stages, effectively addressing local receptive field limitations (Ni et al., 2024). Similarly, Yang et al. (2025) introduced PhenologyNet, which fuses deep features with phenotypic similarity constraints to enhance fine-grained separability (Yang et al., 2025). However, these state-of-the-art approaches often incur high computational costs due to heavy attention blocks or complex auxiliary loss functions. In contrast, AMFR-Net adopts a more targeted strategy: rather than stacking heavy Transformer layers, it employs the lightweight AMSAF module to dynamically recalibrate multi-scale features. This allows the network to achieve comparable or superior discriminability—particularly in distinguishing ambiguous mid-stage transitions—while maintaining a structural efficiency suitable for practical agricultural deployment.

Another significant trend is the development of lightweight architectures for real-time inference and edge deployment. Models such as MobileNet (Liang et al., 2024), EfficientNet (Priya et al., 2024), and YOLOv5 (Ahmad et al., 2022; Tirkey et al., 2023) have been widely adopted for insect and weed detection, offering a balance between computational efficiency and classification accuracy. These compact models have enabled practical applications on embedded systems and mobile platforms, with promising results in reducing pesticide usage and improving field-level monitoring. Additionally, custom adaptations such as Faster-PestNet (Ali et al., 2023) and domain-specific YOLO variants (Song et al., 2023) further demonstrate the flexibility of deep learning models in addressing agricultural constraints.

Complementing ground-level approaches, UAV-based remote sensing has also been employed for crop growth monitoring and yield prediction (Feng et al., 2020; Zhou et al., 2021). These studies leverage multi-sensor imagery to estimate development stages indirectly, though often at coarser spatial resolution and lower temporal frequency compared to ground-level RGB imaging. Moreover, UAV applications face regulatory and operational challenges that hinder scalability in many regions (Cracknell, 2017; Sousa et al., 2022).

Taken together, these studies underscore the growing sophistication of deep learning applications in agriculture. However, most existing efforts prioritize tasks with sharp visual boundaries or high inter-class contrast. In contrast, phenological stage recognition—especially in crops like wheat—poses a unique challenge due to its inherent intra-class ambiguity and gradual morphological shifts. This creates a clear research gap for models capable of capturing fine-grained temporal dynamics under noisy, real-field imaging conditions. Addressing this need requires not only architectural refinement for spatial and scale-aware representation but also a careful balance between model complexity and deployment feasibility. The present study is designed to meet these requirements by introducing a targeted multi-scale feature refinement framework that directly responds to the morphological continuity and visual uncertainty inherent in growth stage identification tasks.

### 1.2 Research gap and contributions

The above literature reveals three gaps for field phenology: (i) limited treatment of intra-class ambiguity and mid-stage sensitivity under real-world noise; (ii) insufficient cross-scale aggregation tailored to stage transitions; and (iii) a lack of deployment-aware evaluation that balances accuracy with model efficiency. In addition, existing SOTA backbones for fine-grained recognition increasingly adopt Transformer-style global reasoning, yet their deployment cost can be prohibitive; thus, there remains a gap for lightweight CNN-based systems that explicitly incorporate long-range and cross-scale interaction mechanisms.

To address these gaps, we propose AMFR-Net, an adaptive multi-scale feature refinement network for wheat growth stage classification from ground-level RGB images. The architecture integrates residual path encoding, cross-scale attention for interaction among feature levels, and weighted aggregation to emphasize context-aware cues in semantically ambiguous regions. Rather than increasing parameters through backbone scaling, the design focuses on targeted refinement to enhance discriminability while keeping the model compact (Hariharan et al., 2018; d’Andrimont et al., 2022; Kordi and Yousefi, 2022). We build a rigorous experimental pipeline on the publicly available CGIAR Wheat Growth Stage dataset with quality-controlled labels and comparative baselines.

Our contributions are threefold.

1. We formulate growth-stage recognition as a high-ambiguity, mid-stage-sensitive classification problem that requires fine-grained spatial reasoning under resource constraints.
2. We develop AMFR-Net, a compact yet expressive model that mitigates inter-stage feature entanglement through hierarchical cross-scale refinement and weighted aggregation.
3. We conduct comprehensive evaluations on CGIAR benchmarks, reporting stage-resolved performance and efficiency suitable for field deployment.

Paper organization. Section 2 details materials and methods, including dataset, preprocessing, model, training, and evaluation. Section 3 presents results with comparisons, ablations, and robustness analyses. Section 4 discusses implications and deployment, and Section 5 concludes.

## 2 Materials and methods

### 2.1 Dataset and preprocessing

#### 2.1.1 Dataset overview

**Figure 1:**
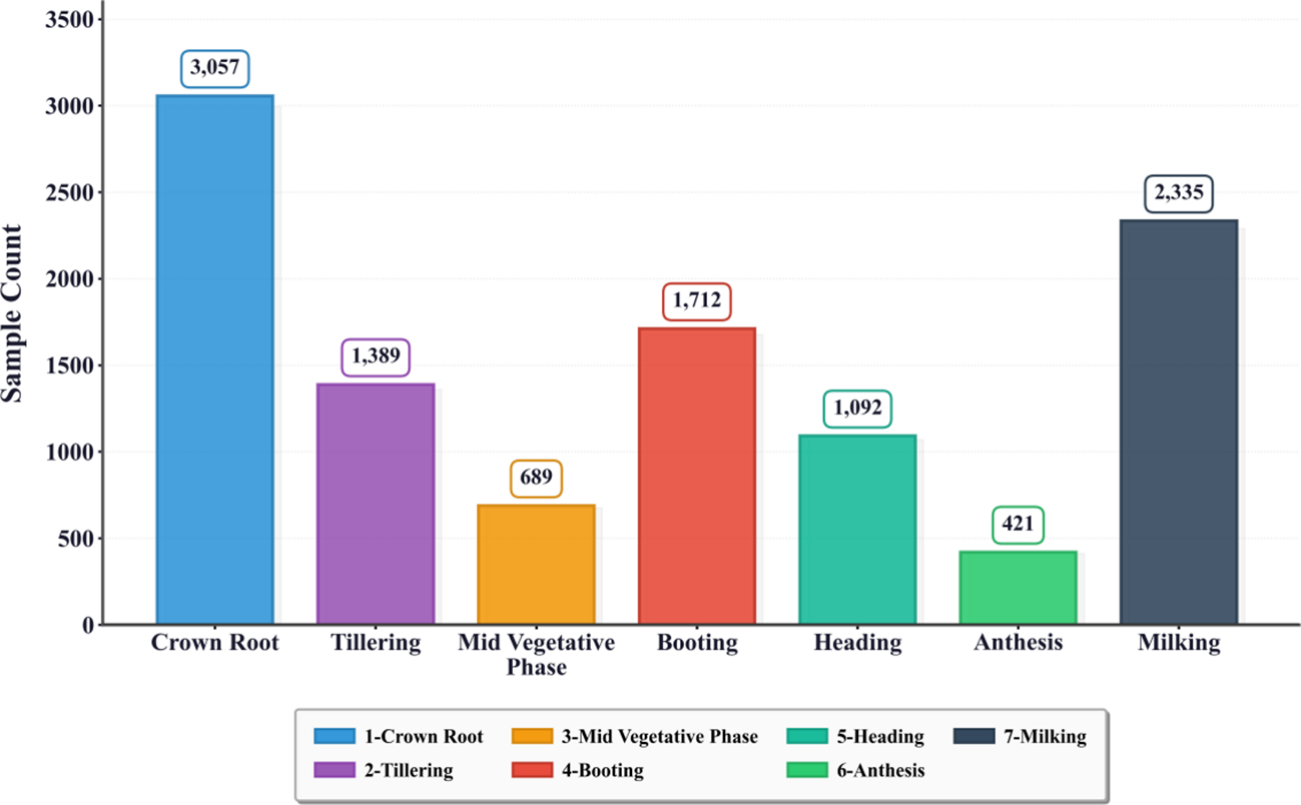
Distribution of all image samples across seven annotated wheat growth stages (label_quality = 1 and 2). This figure shows the number of images per growth stage, grouped by annotation quality (label_quality = 1 vs. 2). It also highlights class imbalance across stages, with some transitional phases underrepresented.

The CGIAR Wheat Growth Stage Challenge dataset (released via the Zindi platform) was collected from repeated field observations over two consecutive Rabi seasons in northwestern India (Hufkens et al., 2019). It contains 14,253 ground-level RGB images annotated into seven growth stages (1–7) and provides an additional label_quality flag distinguishing farmer-reported labels (1) from expert-validated labels (2). Key dataset specifications and the overall stage distribution are summarized in **Table 1**, **Figure 1**, respectively.

**Table 1:** Dataset specification of the CGIAR wheat growth stage dataset used in this study.

Item	Specification
Dataset/source	CGIAR Wheat Growth Stage Challenge dataset (CGIAR Platform for Big Data in Agriculture; Zindi competition portal).
Modality	Ground-level RGB images captured by smartphones.
Study region	Northwestern India: Punjab (Fatehgarh, Ludhiana, Patiala) and Haryana (Fatehabad, Sirsa, Yamunanagar).
Seasons	Two consecutive Rabi (winter) seasons.
Participants/sampling unit	1,685 farmers; one wheat field randomly selected per farmer; repeated observations throughout the season.
Labels	Seven growth stages (1–7): Crown Root Initiation (1), Tillering (2), Mid Vegetative Phase (3), Booting (4), Heading (5), Anthesis (6), Milking (7).
Annotation quality	Train.csv includes label_quality: 2 = expert-validated label; 1 = farmer-reported label (potentially less reliable).
Acquisition protocol	WheatCam Android app with an initial northward-oriented reference photo; subsequent images aligned using a semi-transparent “ghosted” overlay; fixed white balance; reference poles used in part of the first-year collection.
Image preprocessing (provided by host)	Images were screened and anonymized; a trapezoid ROI was automatically delineated via horizon detection (PELT change points on the blue channel) after resizing the image to 640 px in x; non-ROI areas were masked to black.
Subset used in this study	Expert-labeled subset only (label_quality = 2). Stage 1 and Stage 6 were absent in this subset, yielding a 5-stage classification task (Stages 2, 3, 4, 5, 7). Train/test split: stratified 80:20. Model input: images rescaled to 256×256 with geometric and photometric augmentations.

Only the expert-labeled subset (label_quality = 2) is used in our experiments.

#### 2.1.2 Label quality and data augmentation

Ensuring annotation reliability is essential for downstream modeling accuracy, particularly in phenologically sensitive classification tasks. The CGIAR dataset provides two levels of annotation fidelity: expert-labeled samples (label_quality = 2) and farmer-labeled samples (label_quality = 1), each differing substantially in visual clarity and semantic consistency.

**Figure 2:**
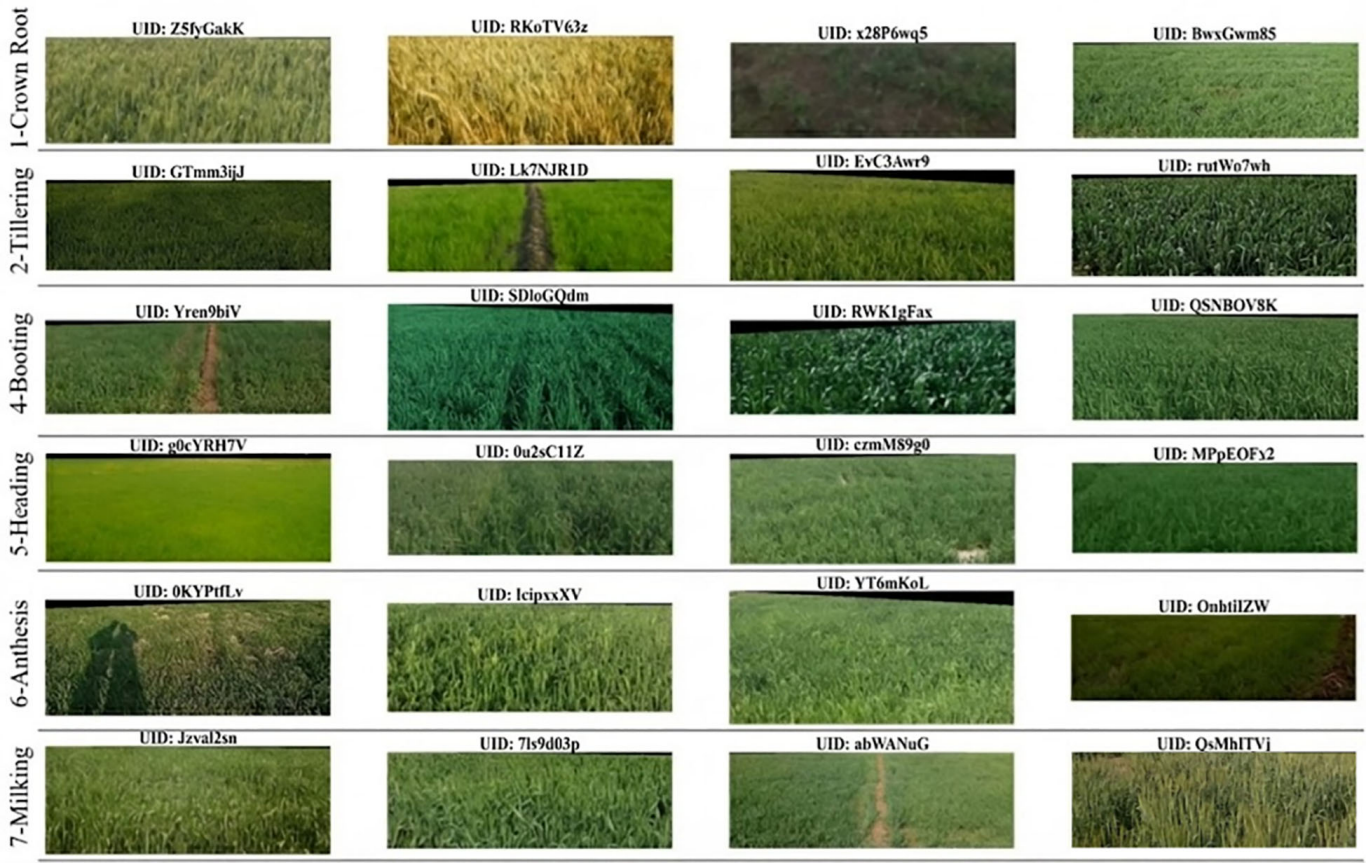
Visual examples from each growth stage annotated by farmers (label_quality = 1), illustrating high intra-class variability and weak boundary clarity. Representative images for the seven stages are shown to highlight large appearance variations within the same class and subtle visual differences between adjacent stages, which complicate stage discrimination.

**Figure 3:**
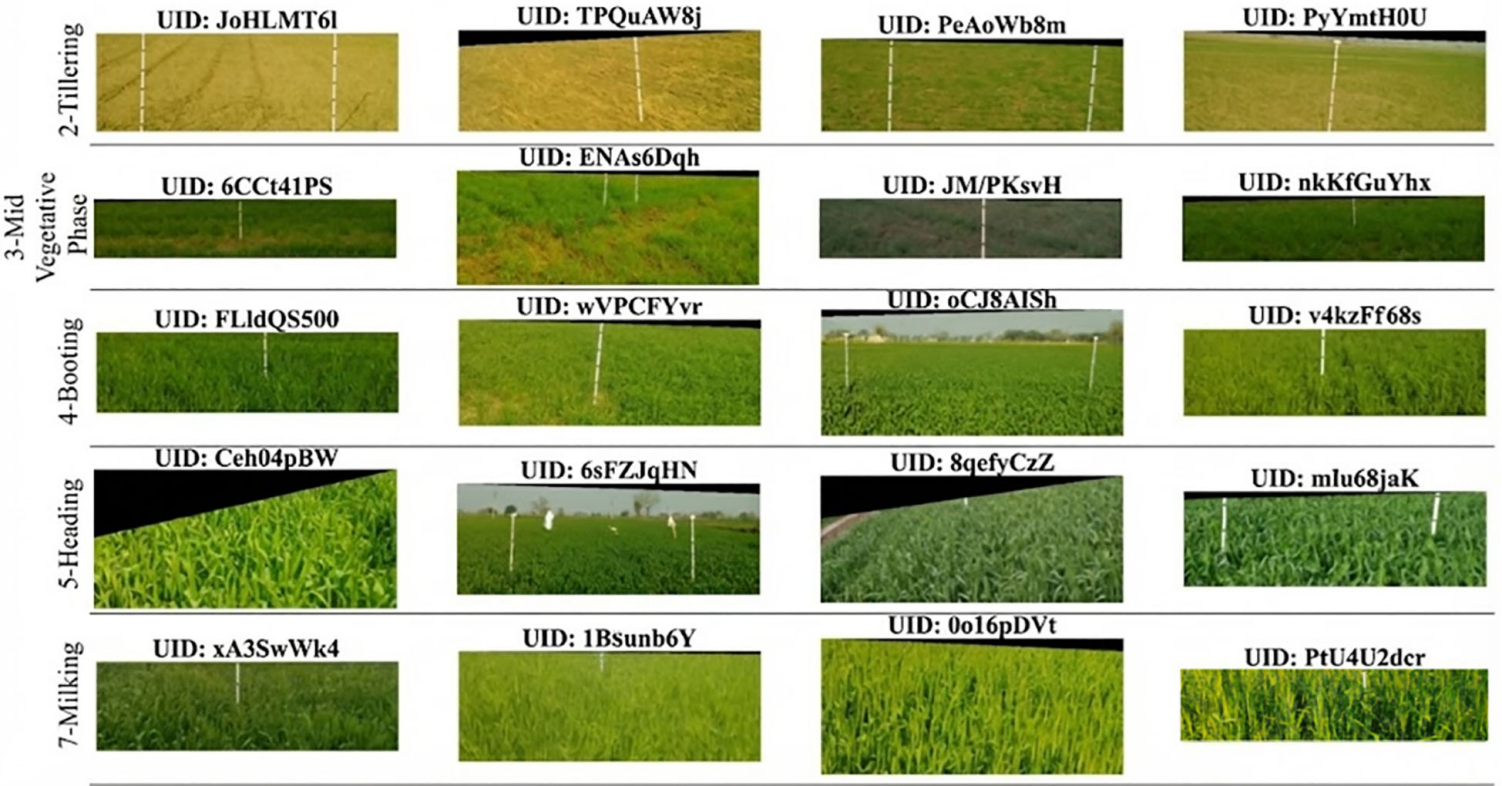
Expert-labeled samples with stage annotations (label_quality = 2) for the five growth stages used in this study. Representative images are shown for Tillering (S2), Mid Vegetative Phase (S3), Booting (S4), Heading (S5), and Milking (S7). These expert-validated samples exhibit more consistent framing and clearer phenological cues than farmer-labeled images, providing more reliable supervision for model training.

A comparative visual inspection first highlights the substantial intra-class variability and weaker inter-stage separability in farmer-labeled samples, which complicates model learning (**Figure 2**). By contrast, expert-annotated images exhibit consistent camera framing and lighting, with prominent phenotypic cues often aided by a vertical reference rod in the scene (**Figure 3**). Given these discrepancies, only the expert-labeled subset was retained for model development.

**Figure 4:**
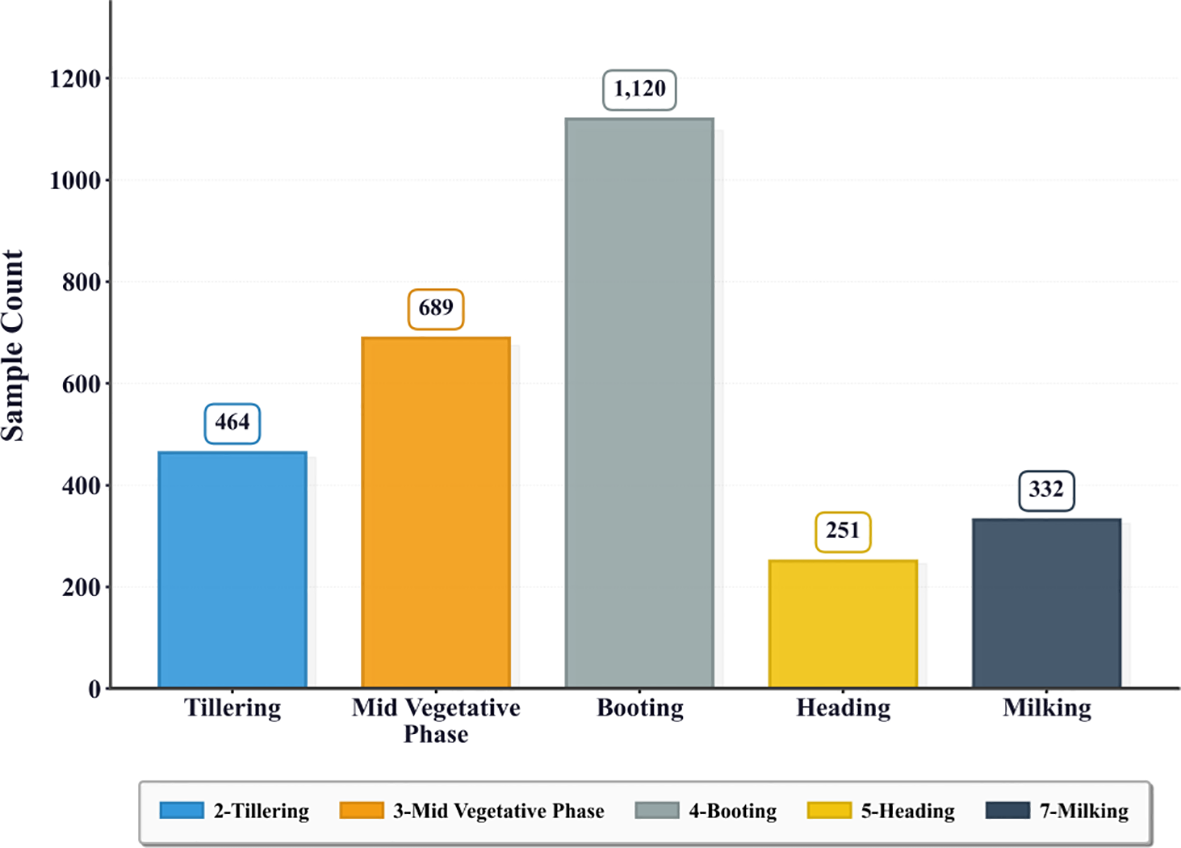
Distribution of training samples across the five retained growth stages (label_quality = 2 only). This figure reports the sample counts for the expert-labeled subset after excluding Stage 1 (Crown Root Initiation) and Stage 6 (Anthesis), leaving five stages for classification. The distribution remains imbalanced, with Booting dominating and Heading having the fewest samples.

However, this decision introduces a constraint: two phenological stages—Crown Root Initiation (Stage 1) and Anthesis (Stage 6)—are not present in the expert-labeled subset, limiting the task to five stages: Tillering (Stage 2), Mid Vegetative Phase (Stage 3), Booting (Stage 4), Heading (Stage 5), and Milking (Stage 7). The refined sample distribution is visualized in **Figure 4**.

To mitigate the effects of reduced sample diversity and enhance model generalization, extensive data augmentation was applied during training. Geometric transformations included random rotation, translation, scaling, horizontal flipping, and region erasure. Photometric augmentations comprised grayscale conversion, contrast and brightness jittering, saturation shifts, and Gaussian noise injection. These techniques help simulate variability in real-world imaging conditions and reduce overfitting risk.

Finally, the dataset was split into training and testing subsets using an 80:20 ratio, with stratified sampling to preserve stage-wise balance across folds.

### 2.2 Task complexity and modeling rationale

The automatic identification of wheat growth stages constitutes a representative fine-grained visual recognition task, where inter-class boundaries are often subtle and intra-class variation remains substantial. Consecutive phenological stages—such as tillering, booting, and heading—exhibit gradual morphological transitions rather than abrupt shifts, making precise discrimination particularly challenging (El Imanni et al., 2022). This intrinsic ambiguity is further compounded by the nature of field-acquired imagery: fluctuating ambient illumination, inconsistent viewing angles, and dynamic environmental conditions (e.g., wind-induced deformation, soil interference) introduce additional sources of noise that undermine model robustness.

While conventional convolutional neural networks (CNNs) have demonstrated effectiveness in standard classification settings, their reliance on rigid receptive fields and limited semantic context restricts their capacity to capture these nuanced stage-specific features. In particular, their performance deteriorates in scenarios where visual cues are subtle, overlapping, or spatially non-salient—a common occurrence in wheat phenotyping tasks where leaf orientation, spike emergence, and canopy density evolve continuously. Standard region-of-interest (ROI) preprocessing can alleviate some spatial inconsistencies, yet residual noise and scale mismatches persist, especially under uncontrolled acquisition protocols.

To address these challenges, we propose a novel architecture—Adaptive Multi-Scale Feature Refinement Network (AMFR-Net)—designed to explicitly model cross-scale morphological cues while enhancing feature saliency through dynamic attention mechanisms. The design is rooted in two key observations: (1) multi-scale representations are essential for capturing both coarse structural patterns and localized fine-grained changes; and (2) adaptive attention mechanisms are critical for suppressing irrelevant context and amplifying discriminative phenotypic signals under noisy visual conditions. The backbone of AMFR-Net builds upon the proven stability of ResNet-101, while integrating an Adaptive Multi-Scale Attention Fusion (AMSAF) module to enable bidirectional interaction across feature hierarchies. This fusion mechanism is specifically tailored to accommodate the gradual evolution of wheat morphology, allowing the network to reconcile fine-scale textural patterns with broader structural semantics in a stage-aware manner.

The subsequent section presents a detailed breakdown of the AMFR-Net architecture and its constituent modules, supported by structural schematics and parameter specifications.

### 2.3 AMFR-Net architecture overview

To effectively capture the complex phenotypic evolution exhibited across wheat growth stages, the proposed AMFR-Net architecture adopts a modular design that systematically integrates deep semantic encoding with adaptive cross-scale feature interaction. The network is organized into four functional components: (1) a shallow feature extraction module that encodes low-level visual patterns; (2) a hierarchical backbone that captures progressively abstract semantics across multiple receptive fields; (3) an Adaptive Multi-Scale Attention Fusion (AMSAF) module that consolidates and refines multi-scale representations via scale-aware attention mechanisms; and (4) a lightweight classification head that performs stage-level prediction based on the aggregated discriminative feature vector.

The network receives as input a three-channel image rescaled to 256×256 resolution. Initial convolutional processing is performed by the shallow feature extraction module, which consists of a 7×7 convolution, batch normalization, ReLU activation, and max pooling operations, yielding a 64×64×64 feature map that encodes primary texture and edge information. This representation is then propagated through the hierarchical feature extraction module, implemented via the standard ResNet-101 architecture from conv2_x to conv5_x. These stacked residual blocks expand the channel depth (256 → 2048) while reducing spatial resolution through strided convolution, producing a set of multi-scale feature maps at {64×64, 32×32, 16×16, 8×8} resolutions.

To overcome the well-documented limitations of static pyramidal aggregation methods and enhance inter-scale contextual modeling, the AMSAF module is introduced downstream of the backbone. It first aligns and transforms intermediate feature maps from multiple stages of the ResNet backbone to a uniform spatial resolution. These aligned maps are then processed through a set of cross-scale interaction blocks (CSIBs), which compute scale-wise attentional calibration using global context vectors derived from all other scales. The refined feature maps are subsequently merged using a content-adaptive Weighted Feature Aggregation (WFA) mechanism, yielding a unified 2048×8×8 representation optimized for stage-level discriminability.

**Figure 5:**
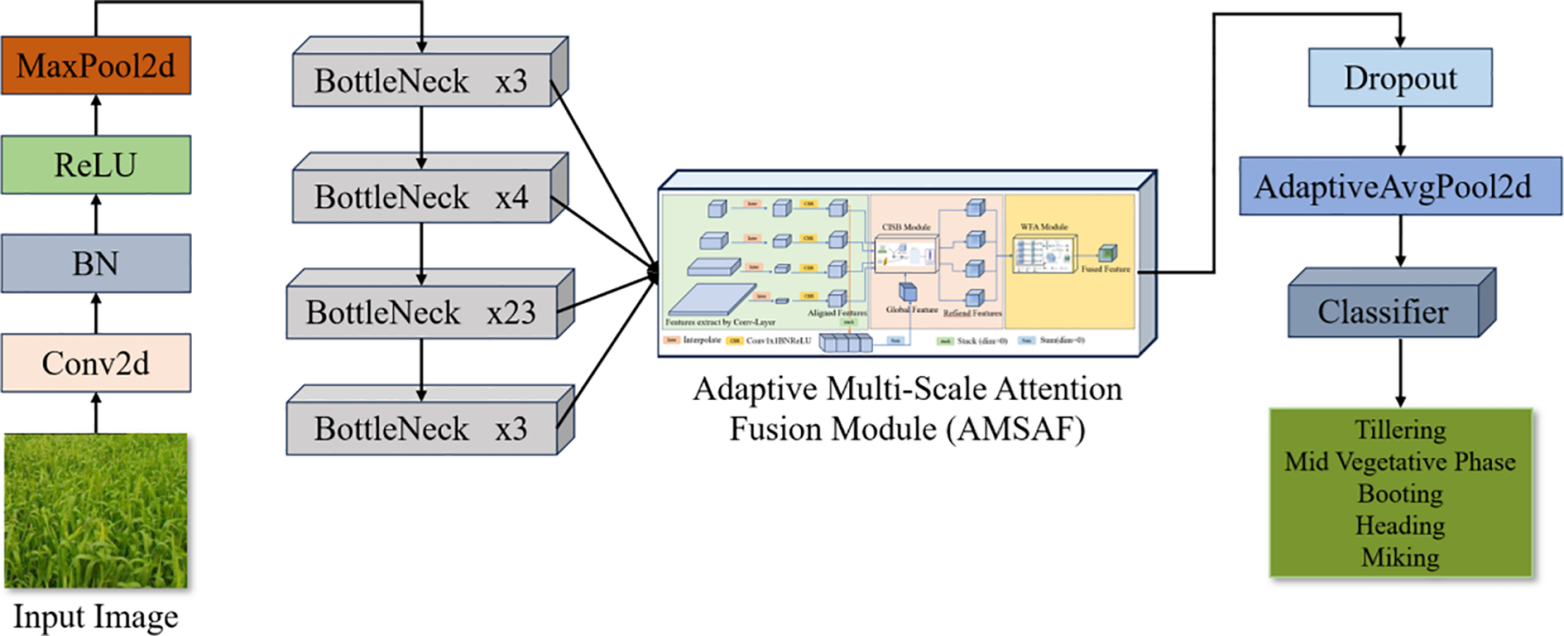
Adaptive multi-scale feature refinement network for wheat growth cycle. The proposed AMFR-Net consists of a shallow feature extractor and a ResNet-101 backbone, followed by the AMSAF module (CSIB + WFA) to refine and fuse multi-scale features. The fused representation is then fed into a lightweight classification head to predict one of five growth stages.

Finally, the classification head performs adaptive average pooling to compress spatial dimensions and applies a dropout regularization layer before feeding the resulting 2048-dimensional vector into a fully connected layer for softmax-based stage classification. The overall architecture is depicted in **Figure 5**, and detailed parameter specifications for each module are provided in **Table 2**.

**Table 2:** Module-level parameters and dimensional specifications of AMFR-Net.

Network layer	Input size	Output size	Parameters
Shallow feature extraction module	3x256x256	64x64x64	9,536
Hierarchical feature extraction module	64x64x64	256x64x64	43,887,872
		512x32x32	
		1024x16x16	
		2048x8x8	
Adaptive Multi-Scale Attention Fusion module	256x64x64	2048x8x8	154,788,236
	512x32x32		
	1028x16x16		
	2048x8x8		
AdaptiveAvgPool2d module	2048x8x8	2048x1x1	--
Classification head module	2048x1x1	5x1	14,341

This table summarizes the input–output feature sizes and parameter counts for each module, from shallow extraction and hierarchical encoding to AMSAF fusion and the final classifier. It highlights how multi-scale fusion changes feature dimensions and contributes to the overall model complexity.

### 2.4 Adaptive multi-scale attention fusion module

**Figure 6:**
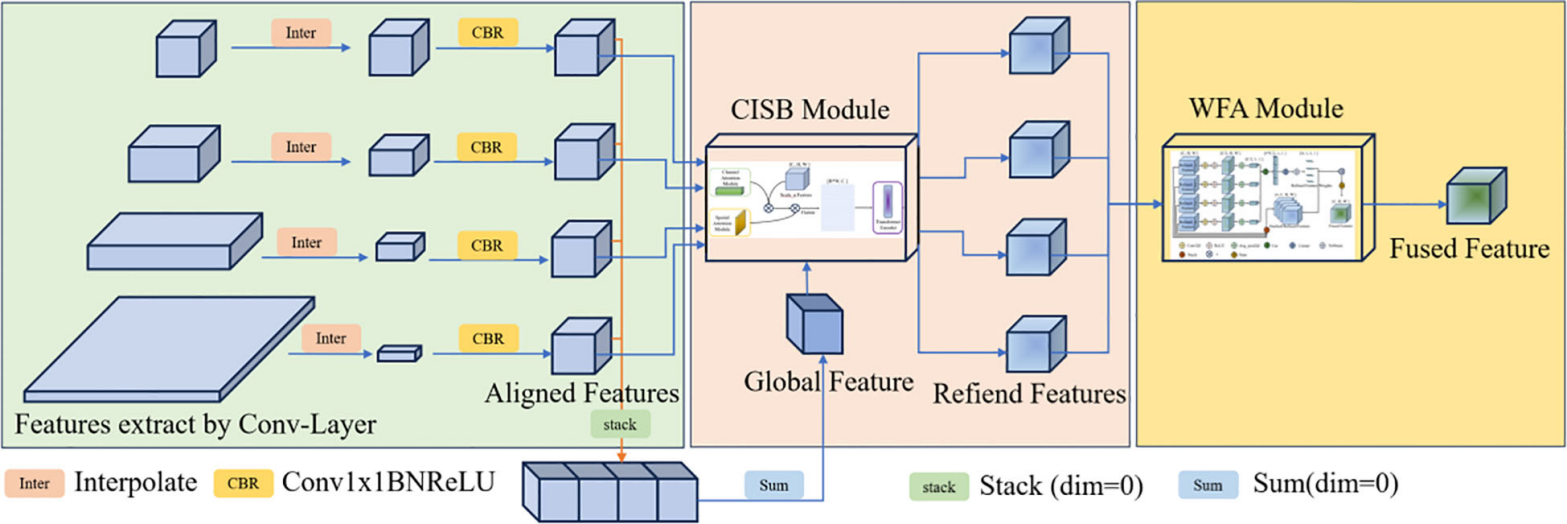
Structure of the adaptive multi-scale attention fusion (AMSAF) module. Multi-scale feature maps from conv2_x–conv5_x are first spatially aligned and channel-normalized, then recalibrated through the Cross-Scale Interaction Block (CSIB) to enable inter-scale information exchange. The refined features are finally integrated by the Weighted Feature Aggregation (WFA) module to generate a single fused representation for classification.

Conventional multi-scale fusion methods, such as Feature Pyramid Networks (FPN) or simple concatenation, typically employ “passive” aggregation. They treat shallow textural features and deep semantic features equally, often leading to semantic misalignment where background noise from low-level layers dilutes the discriminative power of high-level representations. To overcome this, we propose the Adaptive Multi-Scale Attention Fusion (AMSAF) module, which introduces an active recalibration mechanism. As shown in **Figure 6**, AMSAF bridges the semantic gap through two steps: (1) the Cross-Scale Interaction Block (CSIB) aligns features via global context guidance, and (2) the Weighted Feature Aggregation (WFA) performs content-aware dynamic fusion.

As illustrated in **Figure 6**, the module comprises two tightly coupled components: (1) a Cross-Scale Interaction Block (CSIB) that captures long-range contextual dependencies across feature hierarchies, and (2) a Weighted Feature Aggregation (WFA) mechanism that adaptively integrates refined features based on scale-aware confidence scores.

The pipeline begins by extracting four intermediate feature maps from the ResNet-101 backbone: conv2_x to conv5_x. These layers span a semantic depth range from low-level textures to high-level semantics. To enable cross-scale interaction, all feature maps are resampled to match the spatial resolution of the deepest layer (conv5_x), and their channel dimensions are unified via 1×1 convolutions. These normalized representations are then passed to the CSIB for targeted recalibration and subsequently fused by WFA to produce a scale-optimized, discriminative feature embedding.

#### 2.4.1 Cross-scale interaction block

Standard lateral connections often lack sufficient semantic filtering. The CSIB addresses this by utilizing a global context descriptor to actively recalibrate local features. Instead of naively stacking layers, CSIB computes the correlation between the global context and each scale, applying Channel and Spatial Attention to highlight stage-specific regions while suppressing noise. Furthermore, a Transformer encoder is introduced to capture long-range dependencies that limited receptive fields in CNNs miss. This ensures that features from different scales are semantically aligned before fusion, significantly reducing the interference of irrelevant background details compared to direct summation.

**Figure 7:**
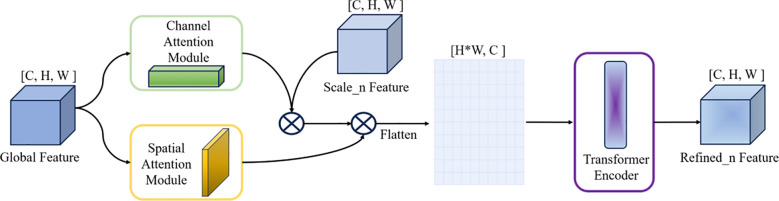
Structure of the cross-scale interaction block (CSIB). The global feature is decomposed into channel and spatial attention maps, which are used to reweight a given scale feature for cross-scale calibration. The reweighted feature is then flattened and passed through a Transformer encoder to model long-range dependencies and output the refined feature for that scale.

As shown in **Figure 7**, CSIB combines the channel and spatial dual attention mechanisms to decouple the global context vector ([C, H, W]) after the multi-scale feature map stacking fusion, and then compresses and excites the feature maps of a specific scale with the channel attention mechanism to generate channel weights and the spatial attention mechanism to calculate the spatial saliency map for spatial-channel decoupling. The decoupled vector is then flattened into a two-dimensional tensor ([H*W, C]) and input into a Transformer encoder. The long-range dependencies of all scales are modeled according to the self-attention layer to generate a scale-global perception vector, adaptively recalibrate the cross-scale dependencies, and the refined features [C, H, W] enhance the discriminability of the semantics of a specific scale. The equation is shown in Equations 1–3.

(1) $$F_{global-CA}=Channel\;Attention\left(F_{global}\right)$$

(2) $$F_{global-SA}=Spatial\;Attention\left(F_{global}\right)$$

(3) $$F_{refined_{n}}=TransformerEncoder(Flatten)(F_{scale}n\;\cdot \;F_{global-CA})\;\cdot \;F_{global-SA}))$$

Among them, ${\text{F}}_{\text{global}}$ is the global eigenvector, ${\text{F}}_{{\text{scale}}_{\text{n}}}$ is the eigenvector of a specific scale, ${\text{F}}_{\text{global}}$ and ${\text{F}}_{{\text{scale}}_{\text{n}}}\in {\text{R}}^{\text{C}\times \text{H}\times \text{W}}$, and ⨂ is the element-by-element multiplication.

#### 2.4.2 Weighted feature aggregation mechanism

**Figure 8:**
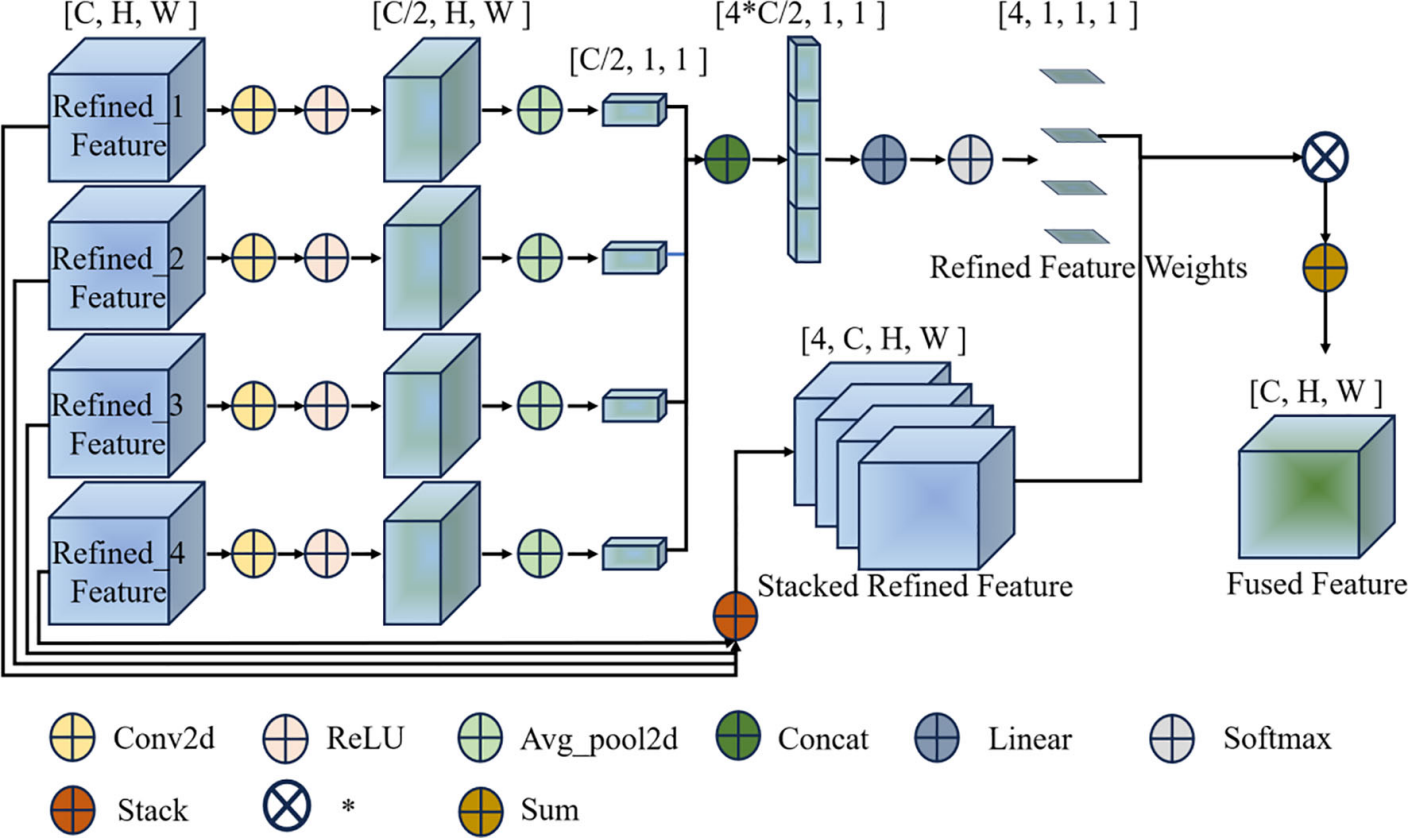
Structure of the weighted feature aggregation (WFA) mechanism. The refined features from four scales are first channel-reduced and globally pooled to generate content-dependent confidence weights via a linear layer and softmax. These weights are then applied to perform a weighted sum over the refined features, producing the final fused feature map.

Different wheat growth stages require information from different scales. Static averaging used in traditional methods fails to capture this variability. The WFA mechanism solves this by learning content-adaptive confidence weights. For each input image, WFA dynamically generates a weight vector W to selectively emphasize the most informative scale. This allows AMFR-Net to adapt its focus instance-by-instance, providing a robust justification for its superior performance in distinguishing morphologically ambiguous stages compared to fixed-weight baselines. As shown in **Figure 8**, its architecture operates in three stages:

(1) Feature Pre-processing: the four refined features ${\text{F}}_{{\text{refined}}_{\text{n}}}\in {\text{R}}^{\text{C}\times \text{H}\times \text{W}}\left(\text{n}=1,2,3,4\right)$ from the CSIB module are standardized through a dedicated 1x1 convolution layer to standardize the number of channels to C/2, as shown in Equation 4:

(4) $${\hat{F}}_{refined_{n}}=ReLU\left(Conv_{1x1}\left(F_{refined_{n}}\right)\right),{\hat{F}}_{refined_{n}}\in R^{\frac{C}{2}\times H\times W}$$

(2) Confidence Weight Generation: the standardized tensor ${\hat{\text{F}}}_{{\text{avgpool}}_{\text{n}}}$ is [C/2,1,1] after global pooling, and then the unified features are connected along the channel dimension for channel stacking (see Equation 5).

(5) $$F_{concat}=Concat\left({\hat{F}}_{avgpool_{1}}+{\hat{F}}_{avgpool_{2}}+{\hat{F}}_{avgpool_{3}}+{\hat{F}}_{avgpool_{4}}\right),F_{concat}\in R^{4*\frac{C}{2}\times 1\times 1}$$

To generate the spatially invariant confidence weights, the concatenated features are fed into a linear layer followed by a softmax function, as formulated in Equation 6.

(6) $$W=Softmax\left(Linear\left(F_{concat}\right)\right)$$

(3) Adaptive Fusion: With the confidence weights $\text{W}=\left[{\text{w}}_{1},{\text{w}}_{2},{\text{w}}_{3},{\text{w}}_{4}\right]$ the feature fusion is computed as illustrated in Equation 7.

(7) $$F_{fused}=\sum_{n=1}^{4}w_{n}\;\cdot \;F_{refined_{n}},\;F_{fused}\in R^{C\times H\times W}$$

### 2.5 Evaluation metrics and protocols

(1) Confusion Matrix: It is a matrix representation that helps evaluate the accuracy of a classification algorithm by describing the relationship between the actual class and the predicted class. It consists of four elements: True Positive (TP); False Positive (FP); True Negative (TN); False Negative (FN). These metrics provide a detailed and nuanced understanding of the performance of AMFR-Net, especially in cases where class imbalance may affect the overall accuracy. **Table 3** shows the confusion matrix used during the training of the AMFR-Net model.

**Table 3:** Confusion matrix used for evaluating AMFR-Net.

Real values	Predicted values	
	Positive	Negative
Positive	TP	FN
Negative	FP	TN

This table defines the four basic outcomes (TP, FP, TN, FN) by comparing predicted labels with ground-truth labels. These terms form the basis for computing accuracy, precision, recall, and F1-score, especially under class-imbalanced settings.

(2) Top-1 Accuracy: Top-1 accuracy is one of the core indicators for evaluating the performance of classification models, and is widely used in multi-category classification tasks. It measures the proportion of the highest probability category in the model’s prediction results that is consistent with the actual label, reflecting the model’s classification ability under the most stringent conditions. Its calculation logic is as follows: For each input sample, the model outputs a probability distribution vector $\text{P}=\left[{\text{p}}_{1},{\text{p}}_{2},\ldots ,{\text{p}}_{\text{N}}\right]$, where N is the total number of categories, and the predicted category $\hat{\text{y}}$ takes the index corresponding to the maximum probability, as defined in Equation 8.

(8) $$\hat{y}=arg\max p_{i}$$

The formula for calculating the Top-1 accuracy is given in Equation 9.

(9) $$Top1\;Accuracy=\;\frac{1}{M}\sum_{j=1}^{M}\;\text{II}\;\left({\hat{y}}_{i}=y_{i}\right)$$

Where M is the total number of test samples and II is the indicator function (1 if the prediction is correct, 0 otherwise).

(3) Precision: The proportion of samples of a certain category that are predicted by the model to be positive examples that are actually positive examples. The calculation formula is as follows (see Equation 10).

(10) $$Precision_{i}=\;\frac{TP_{i}}{TP_{i}+FP_{i}}$$

(4) Accuracy (OvR): In a multi-classification task, the Accuracy (OvR) of a certain category refers to the binary classification accuracy for a specific category when the One-vs-Rest (OvR) strategy is adopted. The core idea is to regard the target category as the positive class and all other categories as the negative class. The recognition ability of the class is evaluated through the binary classification model. The calculation formula is as follows (see Equation 11).

(11) $$Accurcay_{i}=\;\frac{TP_{i}+TN_{i}}{TP_{i}+TN_{i}+FP_{i}+FN_{i}}$$

(5) Recall: Recall is one of the core indicators for evaluating machine learning classification models. It measures the model's coverage of positive samples, i.e., "how many positive samples are correctly identified", as formulated in Equation 12.

(12) $$Recall_{i}=\;\frac{TP_{i}}{TP_{i}+FN_{i}}$$

(6) F1 Score: F1 Score is one of the core indicators for evaluating the performance of classification models. It balances precision and recall through the harmonic mean. It is suitable for processing imbalanced data or scenarios that require comprehensive consideration of false positives and false negatives, as shown in Equation 13.

(13) $$F1\;score=\frac{2\;Precision_{i}\;\times Recall_{i}}{Precision_{i}+Recall_{i}}$$

(7) AUC: In multi-classification tasks, AUC is an important indicator for evaluating the comprehensive performance of the model. The core idea is to convert the multi-classification problem into multiple binary classification problems, and then summarize the results by weighted average of the class sample proportions. The calculation formula is as follows (see Equation 14).

(14) $$AUC_{weighted-ovr}=\sum_{i=1}^{C}{\text{AUC}}_{i}\;\times \text{p}\left({\text{c}}_{i}\right)$$

Where $\text{p}\left({\text{c}}_{\text{i}}\right)$ is the sample proportion of category C.

## 3 Result

### 3.1 Environment and parameter configuration

GPU acceleration was necessary for AMFR-Net because its ~10^8^-parameter scale and multi-branch attention fusion substantially increase both compute and memory consumption during FP32 backpropagation. In practice, several GB are required even for storing parameters, gradients, and optimizer states, and additional memory is needed for multi-scale feature activations. Hence, we trained the model on a GPU-equipped workstation. The hardware and software configurations are described below.

The experiments were conducted on a workstation equipped with an Intel Core™ i7-13700KF processor (5.40 GHz), 32 GB RAM, and an NVIDIA GeForce RTX 4070 GPU with 12 GB of VRAM. The model was implemented in Python 3.13 under the PyTorch 2.7.1 deep learning framework, with CUDA 12.6 enabling GPU computation. The software stack was managed via Anaconda on a Windows 11 64-bit operating system, and code development was conducted using Visual Studio Code (x64-1.102). A summary of the experimental environment is presented in **Table 4**.

**Table 4:** Experimental environment and implementation details.

Category	Parameter
Operating System	Windows 11–64 bits
Development Tool	Vscode-x64-1.102
CPU	Intel Core™ i7-13700KF
GPU	NVIDIA GeForce RTX 4070
Deep Learning Framework	Pytorch 2.7.1
Scripting Language	Python 3.13
RAM	32 GB
VRAM	12 GB

This table summarizes the hardware and software configurations used for training and evaluation, including the operating system, development toolchain, CPU/GPU, and deep learning framework.

This computational setup provided sufficient memory bandwidth and parallel processing capacity to handle the large-scale training data and computational overhead introduced by multi-scale attention mechanisms. In particular, the use of a high-end GPU substantially accelerated both the forward and backward propagation processes, enabling rapid convergence of AMFR-Net without compromising numerical stability. Such an environment ensured that the model could be trained under optimal conditions, facilitating reproducible performance in downstream tasks such as wheat growth stage recognition.

### 3.2 Training procedure and performance evolution

To enable stable and efficient convergence of AMFR-Net, a carefully designed training strategy was adopted, incorporating modern regularization techniques and adaptive learning dynamics. This section outlines the training settings, hyperparameter configuration, and the resulting optimization trajectory.

Given the maturity and robustness of the ResNet-101 backbone used for initial feature extraction, we initialized the training process with a learning rate of $1\times 10^{-4}$, using the AdamW optimizer and setting the weight decay to $1\times 10^{-4}$. The total number of training epochs was set to 100, with a dropout rate of 0.3 introduced after key layers to suppress overfitting during later training stages. Furthermore, an early stopping mechanism with a patience threshold of 20 epochs was applied. When the validation performance plateaued for 10 consecutive epochs, the learning rate was adaptively reduced to 80% of its current value. This dual-stage control—dropout and early stopping—was effective in preserving generalization while allowing the model to fully adapt to complex stage-wise visual variations in wheat growth.

**Figure 9:**
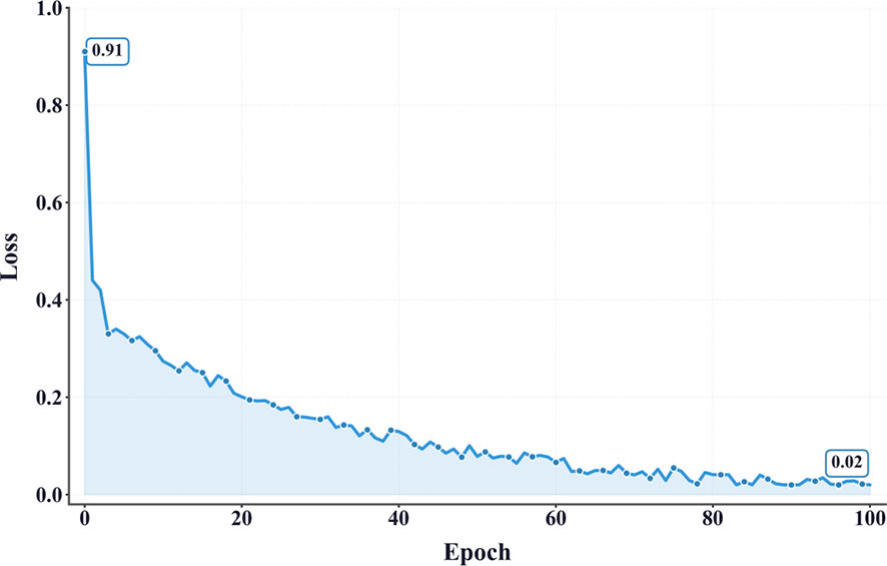
AMFR-Net training loss curve. This figure shows the cross-entropy loss versus epoch, indicating rapid early convergence followed by a gradual plateau as training stabilizes.

As illustrated in **Figure 9**, the training loss exhibits a three-phase trajectory: (1) a rapid decline during the first five epochs, indicating effective initial convergence; (2) a slower yet steady descent between epochs 5 and 40, suggesting continued optimization over complex features; and (3) stabilization beyond epoch 40, followed by early termination around epoch 90, triggered by the early stopping policy. This staged evolution indicates that the model quickly captures low-level patterns, then gradually learns higher-order semantics before entering a plateau phase.

**Figure 10:**
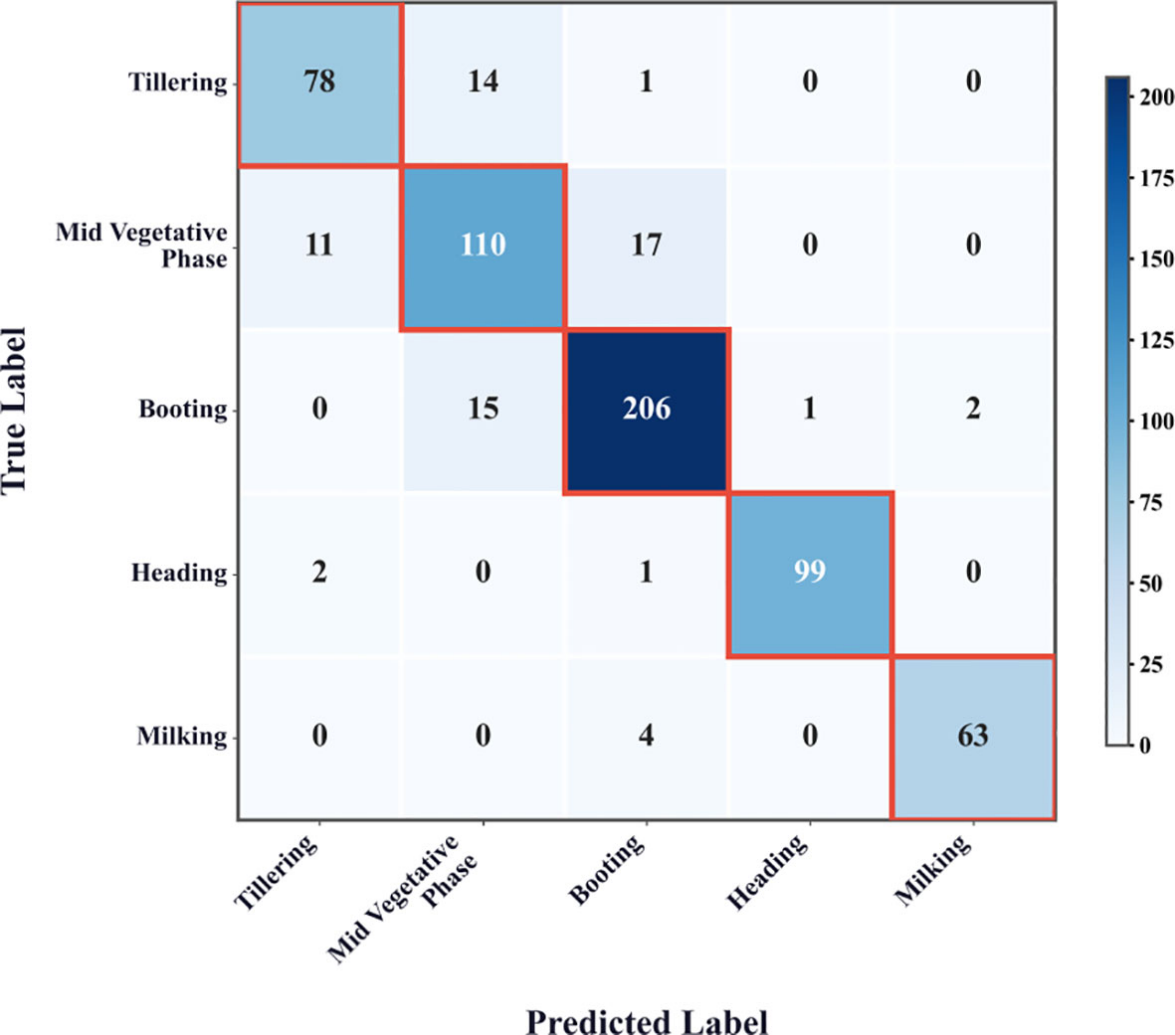
Confusion matrix of AMFR-Net on the test set. Rows denote true labels and columns denote predicted labels, with darker cells indicating higher sample counts.

To assess classification performance, the confusion matrix (**Figure 10**) reveals overall high agreement between predicted and actual growth stages, with the mid-vegetative phase showing the highest rate of misclassification. This suggests that intermediate stages exhibit less distinct phenotypic cues, making them more prone to confusion.

**Figure 11:**
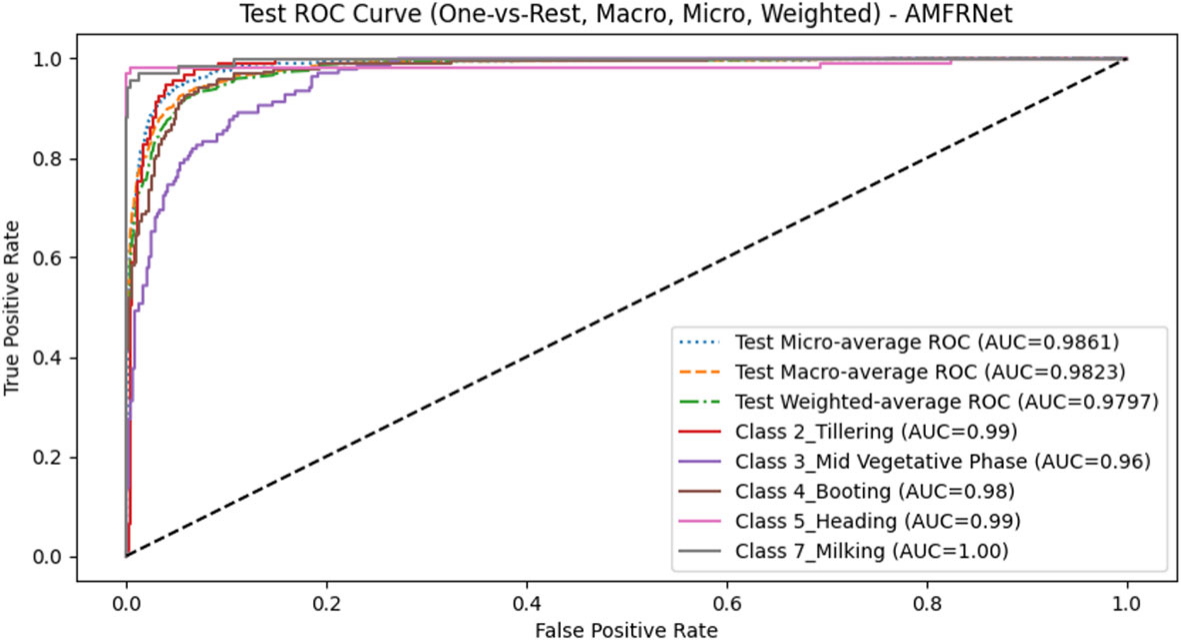
Test-set ROC curves (One-vs-Rest) for five growth stages. ROC curves are plotted for each class under an OvR scheme, together with micro-, macro-, and weighted-average curves.

The ROC curves and AUC scores (**Figure 11**) further validate the model’s discriminative power. Under a One-vs-Rest (OvR) scheme, ROC curves were plotted for each class, and AUC values were computed across micro, macro, and weighted averaging modes. The results demonstrate consistently high separability across categories (AUCs > 0.95 in all modes), reaffirming the robustness of AMFR-Net in multi-class growth stage recognition. However, as noted, the mid-vegetative phase exhibits a comparatively lower AUC, aligning with the misclassification trends observed in the confusion matrix.

While most deep learning models risk either overfitting to transient visual cues or underfitting complex temporal transitions, the training trajectory of AMFR-Net suggests a different pattern. The phased loss evolution, coupled with strategically timed learning rate adjustments and dropout regularization, indicates a stable adaptation path that neither converges prematurely nor stagnates. This balance may prove essential for capturing the subtle visual distinctions among closely aligned growth stages.

### 3.3 Comparative evaluation with benchmark CNNs

To contextualize the performance of AMFR-Net, we conducted a systematic evaluation against several established convolutional architectures widely adopted in image classification tasks, including VGG16, InceptionV3, MobileNetV2, EfficientNet-B0, and DenseNet121. These models offer a diverse spectrum of depth, receptive field configurations, and parameter efficiencies, thus providing a comprehensive baseline for comparison.

**Figure 12:**
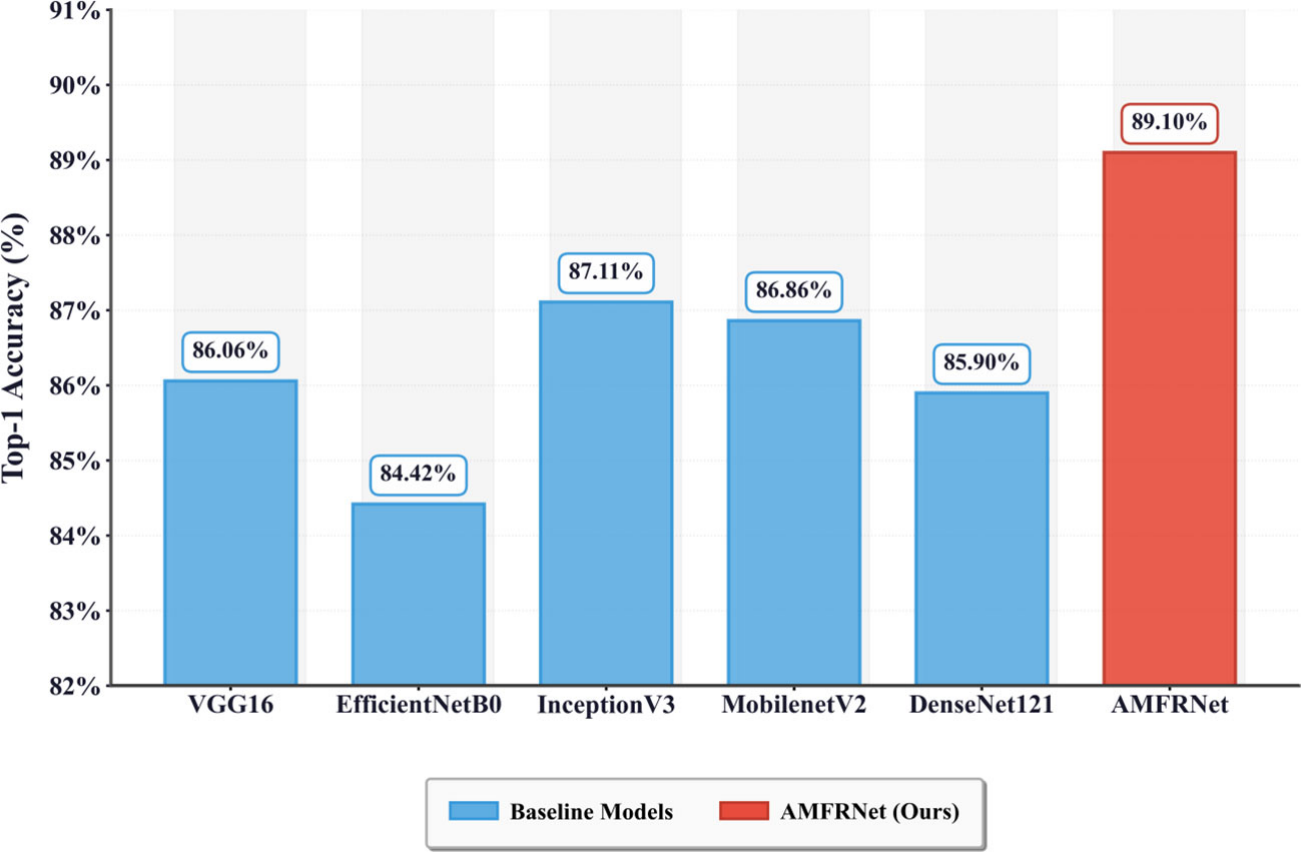
Top-1 accuracy of AMFR-Net and benchmark CNN models on the test set. This figure compares the overall Top-1 accuracy achieved by each model under the same evaluation protocol.

As illustrated in **Figure 12**, **Table 5**, AMFR-Net consistently achieved the highest top-1 accuracy (89.10%), outperforming all benchmark networks. This performance gain may be attributed to its hierarchical fusion structure, which facilitates cross-scale interaction between shallow texture details and deeper semantic abstractions—an advantage particularly relevant for complex agricultural targets where phenological transitions are subtle and class boundaries are weakly defined.

**Table 5:** Top-1 accuracy of AMFR-Net and benchmark CNN models on the test set.

Model	VGG16	EfficientNetB0	InceptionV3	MobilenetV2	DenseNet121	AMFRNet
Top-1 accuracy	86.06%	84.42%	87.11%	86.86%	85.90%	89.10%

This table lists the overall Top-1 accuracy for each compared architecture.

To further dissect performance across specific growth stages, **Table 6** presents stage-wise OvR (One-vs-Rest) classification accuracy. AMFR-Net exhibits superior accuracy in the Mid Vegetative Phase (90.87%), Booting (93.43%), Heading (99.36%), and Milking (99.04%) stages. Notably, these phases are often visually confounded due to morphological similarity or canopy occlusion, suggesting that AMFR-Net’s attention-guided multi-scale fusion offers improved discriminability under inter-class ambiguity.

**Table 6:** One-vs-Rest (OvR) accuracy of AMFR-Net and benchmark CNN models for each growth stage.

Growth stage	VGG16	EfficientNetB0	InceptionV3	MobilenetV2	DenseNet121	AMFRNet
Tillering	95.35%	95.35%	95.99%	95.67%	95.35%	95.51%
Mid Vegetative Phase	88.62%	88.62%	89.58%	89.58%	88.62%	90.87%
Booting	91.03%	89.42%	91.51%	91.67%	90.71%	93.43%
Heading	99.04%	97.76%	99.04%	98.88%	99.20%	99.36%
Milking	98.08%	96.79%	98.24%	97.92%	97.92%	99.04%

This table reports per-stage OvR accuracy for the five retained growth stages.

Complementary evaluation through F1-score (**Table 7**) and recall (**Table 8**) confirms the robustness of AMFR-Net, particularly in imbalanced or visually ambiguous classes. For example, in the Booting stage, AMFR-Net achieves both the highest F1-score (90.95%) and recall (91.96%), outperforming even deeper networks such as DenseNet121. This indicates a stable generalization capability that persists across both precision- and recall-sensitive metrics.

**Table 7:** Per-stage F1-score of AMFR-Net and benchmark CNN models.

Growth stage	VGG16	EfficientNetB0	InceptionV3	MobilenetV2	DenseNet121	AMFRNet
Tillering	83.80%	83.04%	88.63%	85.41%	84.15%	84.78%
Mid Vegetative Phase	75.60%	74.73%	76.01%	76.70%	74.91%	79.42%
Booting	87.50%	85.65%	88.30%	88.50%	87.05%	90.95%
Heading	97.00%	92.86%	97.06%	96.52%	97.56%	98.02%
Milking	90.77%	85.71%	97.13%	90.08%	89.92%	95.45%

This table summarizes class-wise F1-scores, reflecting the balance between precision and recall for each stage.

**Table 8:** Per-stage recall of AMFR-Net and benchmark CNN models.

Growth stage	VGG16	EfficientNetB0	InceptionV3	MobilenetV2	DenseNet121	AMFRNet
Tillering	80.65%	76.34%	87.10%	84.95%	82.80%	83.87%
Mid Vegetative Phase	71.90%	76.09%	74.64%	75.89%	73.10%	79.71%
Booting	87.50%	87.95%	89.29%	89.29%	87.05%	91.96%
Heading	95.10%	89.22%	97.06%	95.10%	98.04%	97.06%
Milking	88.06%	89.55%	91.04%	88.06%	86.57%	94.03%

This table reports class-wise recall values for each growth stage.

What becomes evident is not merely the superiority of AMFR-Net in aggregate metrics, but also its resilience in stage-wise detection where conventional models tend to degrade. Could this suggest that conventional architectures, while deep, still lack the structural adaptability required to capture intra-class variation over time? The following ablation experiments attempt to decouple this hypothesis by isolating the role of adaptive attention fusion in AMFR-Net.

### 3.4 Ablation experiment comparison

To isolate the contribution of the proposed Adaptive Multi-Scale Attention Fusion (AMSAF) module, a series of ablation studies were conducted by systematically modifying key architectural components of AMFR-Net. Rather than merely benchmarking performance, these experiments were designed to probe a deeper question: To what extent does attention reweighting, spatial context, and hierarchical fusion individually shape the network’s ability to discriminate phenologically adjacent wheat stages?

**Table 9**, **Figure 13** present performance comparisons across four architectural variants:

**Table 9:** AMFR-Net ablation experiment performance comparison.

Architecture variant	Top-1 accuracy	F1-score	Recall	AUC
Backbone+ Standard classification head	86.18%	86.19%	86.22%	97.10%
Backbone+Channel Attention	87.70%	87.66%	87.66%	97.51%
Backbone+ Spatial Attention	87.31%	87.26%	87.34%	97.82%
Backbone+ Multi-scale fusion	86.96%	86.33%	86.22%	97.14%
Backbone+ AMSAF(AMFR-Net)	89.16%	89.10%	89.12%	97.88%

This table reports Top-1 accuracy, F1-score, recall, and AUC for each ablation variant (baseline, channel attention, spatial attention, multi-scale fusion, and full AMSAF).

**Figure 13:**
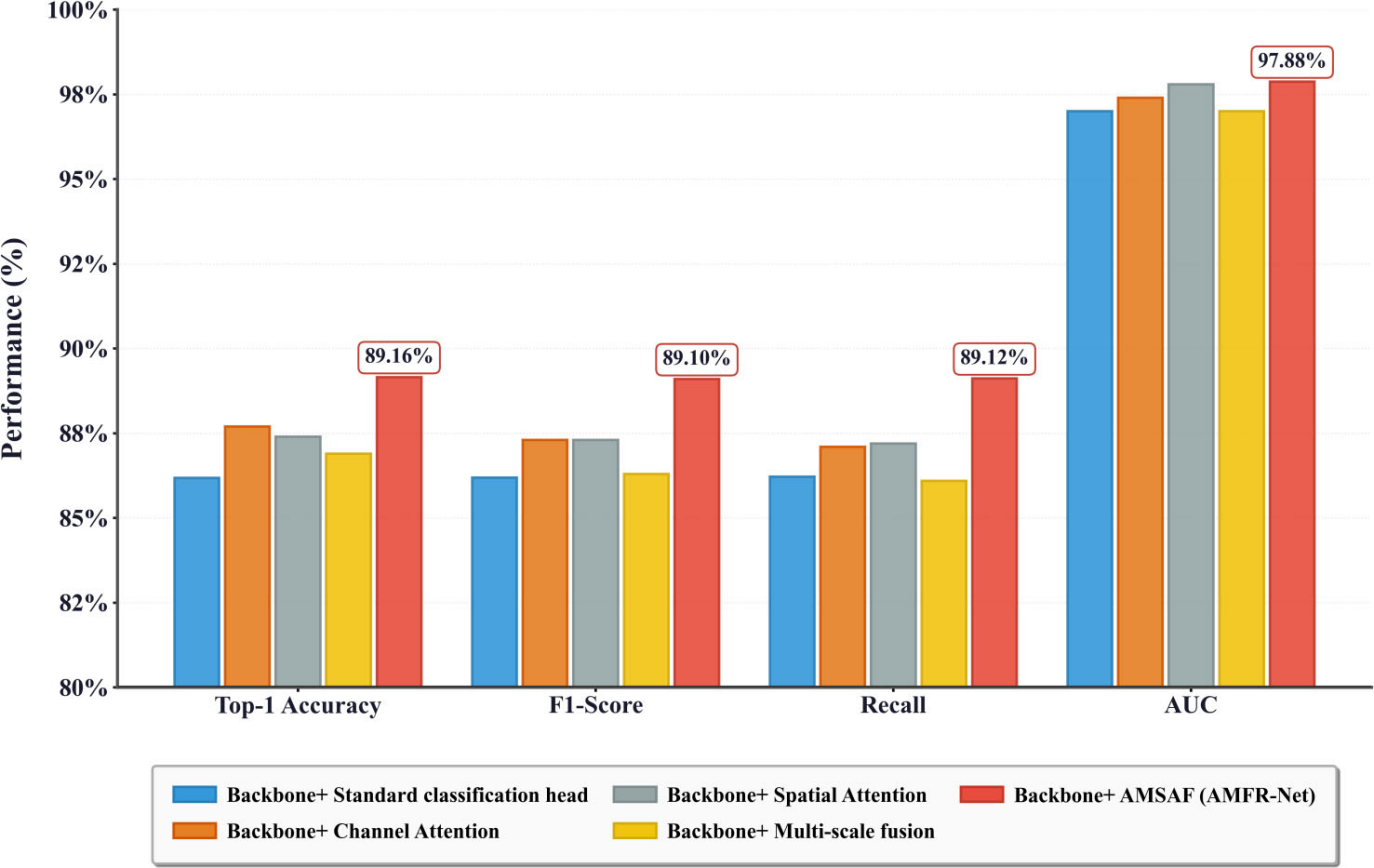
AMFR-Net ablation experiment performance comparison. This figure compares Top-1 accuracy, F1-score, recall, and AUC for the baseline backbone and different attention/fusion variants, including the full AMSAF module.

1. The Baseline model, which uses the same ResNet101 backbone with a standard global average pooling and fully connected head, offers a performance floor (Top-1 accuracy: 86.18%). Its limited ability to capture localized phenotypic cues or cross-scale patterns underscores the insufficiency of global representations alone.
2. Variant 1 and Variant 2, incorporating channel-wise and spatial attention respectively, show moderate gains across all metrics (Top-1 accuracy: 87.70% and 87.31%). These improvements suggest that dimension-specific reweighting enhances feature saliency, yet remains spatially or semantically constrained.
3. Variant 3, employing a simple multi-scale fusion strategy akin to FPN, yields only marginal benefits over the baseline (Top-1 accuracy: 86.96%), indicating that scale diversity alone is not inherently beneficial without adaptive coordination across hierarchies.

In contrast, AMFR-Net, which integrates the full AMSAF module, achieves the best performance across all evaluated indicators (Top-1 accuracy: 89.10%, F1: 89.10%, Recall: 89.12%, AUC: 97.88%). This outcome suggests that the fusion of channel and spatial attention, when modulated across multiple semantic levels via adaptive weighting, substantially enhances discriminative representation learning—particularly in tasks involving fine-grained temporal variation.

These results collectively demonstrate that while single-dimensional attention (either channel or spatial) and naive multi-scale fusion can bring moderate improvements, their effects remain limited when applied in isolation. The full AMSAF module achieves consistent gains across all evaluation metrics by jointly capturing cross-scale semantics and enhancing spatial-channel interactions. This indicates that its strength lies not in any individual component, but in the coordinated integration of attention and hierarchical fusion, which effectively enhances the model’s ability to distinguish subtle phenological differences across growth stages.

In addition to accuracy-oriented metrics, we further report the efficiency implications of introducing different attention/fusion components. **Table 10** summarizes the parameter scale, estimated computational cost, and inference latency of each ablation variant under the same input resolution and hardware setting. As shown in **Table 10**, lightweight channel/spatial attention introduces only a minor overhead compared with the baseline, whereas the full AMSAF module yields the most significant performance gain at a higher computational cost, thereby clarifying the practical accuracy–efficiency trade-off of AMFR-Net.

**Table 10:** Efficiency comparison of attention/fusion variants (input: 256×256).

Variant	Added component	Top-1 Acc. (%)	Params (M)	FLOPs (G)	Inference latency (ms/img)
Backbone + standard head (Baseline)	None	86.18	43.9	≈10.3	≈12.0
Backbone + Channel Attention (Variant 1)	Channel attention	87.7	44.3	≈10.6	≈12.7
Backbone + Spatial Attention (Variant 2)	Spatial attention	87.31	44.1	≈10.7	≈12.9
Backbone + Multi-scale fusion (Variant 3)	FPN-like fusion	86.96	47.4	≈12.2	≈15.0
Backbone + AMSAF (AMFR-Net)	Cross-scale attention + WFA	89.16	198.7	≈18.0	≈22.5

## 4 Discussion

### 4.1 Morphological ambiguity and feature representation bottlenecks

Despite the overall superiority of AMFR-Net across multiple performance metrics, the model’s classification accuracy notably declines when tasked with identifying transitional phenological stages—most prominently, the Mid Vegetative Phase. This performance dip, evidenced by lower F1-scores and recall values, reveals a structural challenge inherent not just to AMFR-Net, but to visual phenotyping tasks broadly: the difficulty of isolating semantically meaningful features in morphologically ambiguous growth intervals.

Unlike classical object recognition tasks—where category boundaries are visually discrete and anchored in shape, texture, or color—wheat growth stages evolve continuously. During transitional phases such as Mid Vegetative or Booting, the visual appearance of wheat plants lacks stable anchors: canopy density increases without marked spatial differentiation, leaf architecture overlaps chaotically, and color gradients remain subdued due to homogeneity in chlorophyll concentration (Batunacun et al., 2021; Abdi et al., 2024; Yang et al., 2025). These weak cues reduce intra-class cohesion while simultaneously increasing inter-class confusion, undermining the discriminative power of even advanced multi-scale architectures.

From a representational perspective, such ambiguity exposes a fundamental bottleneck in attention-driven feature extraction: the lack of sufficient saliency contrast to guide meaningful reweighting (Batista et al., 2023). In the AMFR-Net framework, the AMSAF module attempts to resolve this by fusing features from multiple semantic depths via the Cross-Scale Interaction Block (CSIB) and Weighted Feature Aggregation (WFA). However, in the absence of strong low- or mid-level anchors, the attention maps generated by CSIB may default to spatially diffuse or semantically neutral activations—failing to amplify the subtle cues necessary for stage separation.

Moreover, the spatial uniformity of RGB imagery under fixed-scale input exacerbates this problem. Unlike hyperspectral or multispectral sources, RGB sensors are unable to capture biochemical changes—such as pigment transitions or cellular structure development—that precede or accompany morphological shifts. As a result, the model is forced to rely solely on visual texture and form, which are inherently weak or noisy during intermediate stages. This limitation suggests that the performance bottleneck lies not in the network depth or attention design per se, but in the insufficient discriminative energy present in the input domain during phenological transitions (Latif, 2019).

Addressing this challenge calls for a rethinking of the representational pipeline, particularly for ambiguous growth phases. One direction is the integration of domain-specific priors—such as stage-aware positional encoding or development-informed spatial masks—that constrain attention to biologically relevant regions (e.g., spikelets, collar regions, or flag leaves). Another promising avenue is to reformulate the classification task into a regression or ordinal prediction problem, capturing stage progression as a continuum rather than a discrete set of classes. This would allow the network to leverage soft transitions and hierarchical relationships between stages, mitigating over-segmentation risks.

In summary, the observed performance degradation in morphologically ambiguous stages is not a failure of architecture but a reflection of the semantic sparsity and continuity of biological processes. It highlights the need for more expressive input representations, more context-aware feature calibration, and possibly a paradigm shift in task formulation—from static classification to dynamic phenological modeling. As agricultural phenotyping continues to evolve, addressing such bottlenecks will be critical for improving not just model accuracy, but also biological fidelity and agronomic utility.

### 4.2 Annotation fidelity and ecological validity tradeoff

The decision to train AMFR-Net solely on expert-annotated images enhances label consistency and improves the reliability of supervised learning. However, this choice also introduces a structural constraint: a reduced exposure to the visual diversity and noise characteristics present in real-world agricultural imagery. This tradeoff between annotation fidelity and ecological validity raises concerns about the model’s generalization capacity when deployed beyond controlled settings.

Expert-labeled samples typically exhibit standardized framing, lighting, and clearly distinguishable phenotypic features, making them well-suited for training and evaluation in experimental pipelines. Yet, field-deployed systems are expected to operate under far less predictable conditions—varying illumination, camera angles, and background interference—many of which are better represented in farmer-labeled or crowdsourced imagery (Htitiou et al., 2021). By excluding these less consistent samples, the model is optimized for internal validity but potentially weakened in external robustness.

This issue is further complicated by the incomplete stage coverage of the expert-labeled dataset. Specifically, the absence of Crown Root Initiation and Anthesis stages disrupts the phenological sequence and narrows the model’s understanding of stage transitions. As a result, the network may struggle to infer contextual progression, particularly in mid-stage classifications where inter-class boundaries are already ambiguous.

More broadly, this reflects a limitation of standard supervised learning setups in agricultural tasks: annotations are treated as fixed ground truths, while in reality, phenological boundaries can be gradual and subject to interpretation—even among experts. Rigid reliance on clean labels may therefore fail to capture the variability necessary for robust decision-making in dynamic field environments.

To address this, future efforts could incorporate semi-supervised learning approaches that retain expert labels as anchors but leverage large-scale, low-fidelity data to introduce variability and noise patterns into training. Methods such as pseudo-labeling, consistency regularization, or confidence-based sample selection could support this integration without compromising overall model reliability (Adhikari et al., 2025).

In addition, teacher–student frameworks may serve as a middle ground, where a model trained on expert labels is used to generate soft labels for farmer-labeled samples, thus expanding the dataset without direct manual curation. This could also help reintroduce the missing stages by estimating their likely positions in the feature space, improving the network’s capacity to model full-cycle phenology.

Ultimately, achieving a balance between annotation quality and ecological representativeness is essential for developing phenotyping models that perform consistently across both curated datasets and real deployment scenarios. Rather than treating these two goals as mutually exclusive, a more flexible training pipeline—capable of integrating diverse annotations with appropriate safeguards—will be key to improving model reliability in practical applications.

### 4.3 Toward deployment-aware and explainable phenotyping models

While AMFR-Net achieves strong performance under curated experimental conditions, its applicability in operational agricultural contexts depends on more than accuracy alone. Real-world deployment introduces a distinct set of constraints—ranging from device limitations to environmental variability—that challenge model stability, interpretability, and usability. Addressing these factors is essential for moving from controlled validation to reliable field use.

Deployment Feasibility and Modular Optimization. As quantified in **Table 10**, AMFR-Net prioritizes classification precision, utilizing a ResNet-101 backbone that results in a parameter count of 198.7M and an inference latency of ≈22.5 ms per image. While this footprint is computationally intensive for entry-level smartphones, it remains well within the operational capabilities of high-end edge computing platforms (e.g., NVIDIA Jetson Orin) used in autonomous farm machinery. More importantly, the proposed AMSAF module is structurally modular. For strictly resource-constrained scenarios, the heavy backbone can be seamlessly replaced with lightweight alternatives such as MobileNet or GhostNet. This flexibility allows practitioners to trade marginal accuracy drops for significant gains in speed and memory efficiency, tailoring the model to specific hardware constraints (Fiorentini et al., 2024).

In addition to computational considerations, visual and environmental variability presents a persistent challenge. Field conditions often introduce occlusion from overlapping crops, inconsistent framing due to manual camera operation, and noise from soil background or fluctuating illumination. Although the AMSAF module enhances multi-scale adaptability, it is not specifically designed to account for such noise sources. Adaptive preprocessing pipelines—such as illumination normalization or dynamic cropping—and more robust spatial attention mechanisms may further improve model resilience under these conditions.

Another important but underutilized aspect of the current framework is interpretability. While AMFR-Net employs attention-based modules that inherently produce activation maps, these outputs are not directly leveraged for model explanation. In practical settings, particularly for agronomists and end-users unfamiliar with deep learning systems, the ability to visualize model focus areas—for example, through saliency maps or stage-specific attention heatmaps—can greatly enhance trust and facilitate validation. Such visual feedback may also assist in identifying systematic errors or recurring misclassifications tied to specific growth conditions or image characteristics.

Moreover, explainable outputs can play a role in human–AI collaboration. For instance, attention visualizations could help agricultural practitioners verify or correct model predictions in ambiguous cases, supporting semi-automated annotation or decision support workflows. This, in turn, could provide additional labeled data for future model refinement in a feedback loop.

Finally, there is growing interest in extending phenotyping models beyond single-modal RGB input. Incorporating multi-modal data sources—such as NDVI indices, environmental sensors, or temporal image sequences—could provide more stable features across growth stages and help the model disambiguate phenotypically similar intervals. While this study focuses on RGB-based classification, the current architecture could be adapted to handle additional channels or fused modalities with relatively minor structural adjustments.

In summary, ensuring deployment readiness for phenotyping models like AMFR-Net involves multiple layers of refinement: architectural simplification for resource-constrained environments, robustness to environmental noise, integration of interpretability tools, and eventual expansion toward multi-modal sensing. These aspects are essential for closing the gap between high-performance models in research settings and reliable, user-friendly systems in agricultural practice.

## 5 Conclusion

In this study, we present AMFR-Net, an Adaptive Multi-Scale Feature Refinement Network designed to tackle the challenging task of wheat growth stage identification under real-field conditions. By integrating a ResNet-101 backbone with the novel Adaptive Multi-Scale Attention Fusion (AMSAF) module, the proposed architecture effectively captures both fine-grained morphological cues and long-range contextual dependencies. Extensive experiments demonstrate that AMFR-Net achieves superior performance across all major metrics, including a Top-1 accuracy of 89.10%, macro-average F1-score of 89.10%, and AUC of 97.88%, consistently outperforming competitive benchmarks such as InceptionV3, DenseNet121, and EfficientNet-B0.

More importantly, AMFR-Net exhibits robust stage-wise discriminability, especially in phenologically adjacent stages such as Booting and Mid Vegetative Phase, where traditional CNNs tend to degrade. Ablation experiments further validate that the combined design of cross-scale interaction and attention reweighting significantly enhances representational quality. The model also maintains stable generalization under data imbalance and fine-class granularity, as reflected in OvR, precision, recall, and F1-score evaluations across all five retained stages.

While the current implementation relies on expert-annotated imagery and omits two stages due to label availability, this design choice ensures high intra-class consistency and training stability. The model’s slightly lower performance in transitional phases like Mid Vegetative Phase reflects inherent phenotypic ambiguity rather than architectural deficiencies. These observations suggest potential avenues for improvement, such as incorporating multispectral data, leveraging time-series imaging, or introducing light-weight attention calibration for deployment under uncontrolled field environments.

Overall, the proposed AMFR-Net offers a scalable, accurate, and interpretable solution for *in-situ* wheat growth monitoring, bridging the gap between controlled experimental validation and real-world agricultural application. Future work will focus on restoring full-stage coverage, enhancing label diversity through weak supervision, and embedding attention visualization tools to further support agronomic interpretation and trust in model decisions.

## Data Availability

Publicly available datasets were analyzed in this study. This data can be found here: Dataset name: CGIAR Wheat Growth Stage Challenge Primary repository: Zindi (official competition page) Direct link: https://zindi.africa/competitions/cgiar-wheat-growth-stage-challengeAccession/DOI: Not assigned (competition-hosted download).
